# An Esthetic Approach for Rehabilitation of Long-Span Edentulous Arch Using Artificial Intelligence

**DOI:** 10.7759/cureus.38683

**Published:** 2023-05-07

**Authors:** K. Sheela Kumari, Anusha K.S.

**Affiliations:** 1 Prosthodontics, Priyadarshini Dental College and Hospital, Chennai, IND; 2 Prosthodontics, Sri Ramachandra Institute of Higher Education and Research, Chennai, IND

**Keywords:** broadrick occlusal plane analysis, removable dentures, precision, distal extension, centric record, chatgpt, attachments

## Abstract

Successful full-mouth rehabilitation requires contemporary and advanced treatment planning, especially in distal extension cases. Multiple treatment modalities are available in those cases. Treatment outcome in these patients remains challenging. Though implants are one of the treatment options in such scenarios, fixed removable partial dentures with precision attachments are the best treatment options for patients who cannot afford expensive treatment. We have made an attempt to describe a case report of a long-span edentulous arch by incorporating the ideas and information received from Chat Generative Pre-trained Transformer (GPT).

## Introduction

Rehabilitation of a partially edentulous arch remains challenging in providing a satisfactory esthetic and functional restoration. The replacement of unilateral or bilateral posterior edentulous segments involves diverse techniques and can be done with various contemporary and conventional modes of treatment options. In such cases, it is not possible to advise fixed partial denture because of the absence of distal abutment. Implant-supported restoration can be planned in these cases, but implants are contraindicated in instances such as insufficient bone in the edentulous area and economic feasibility. In such cases, cast partial dentures and precision attachments are preferred.

For best outcome in prosthodontic treatments (either removable partial denture, fixed partial denture, or cast partial denture with attachments), the prosthesis should have good retention and stability, and it should satisfy the functional and esthetic requirements of the patient. Various studies were done and have shown the survival rate of 83.35% for five years, of 67.3% up to 15 years, and 50% up to 20 years [1,2]. This paper describes the full mouth rehabilitation of a patient with maxillary bilateral distal extension Kennedy's class I situation, which is restored by a cast partial denture retained using extra coronal precision attachment, and the mandibular arch is restored with conventional fixed partial denture.

We wrote this article by taking into consideration of certain information received from Chat Generative Pre-trained Transformer (GPT). It is one of the recently evolving new artificial intelligence (AI)-based technology that has created a chaotic revolutionary effect in engineering as well as in medical fields. It was described to be evolutionary chatbots accessed by the GPT. It allows the candidate to interact in a more conversational manner to a text-based prompts. It can be synonymized as Glossary of Preserved Information Transformers in various fields.

## Case presentation

**Figure 1:**
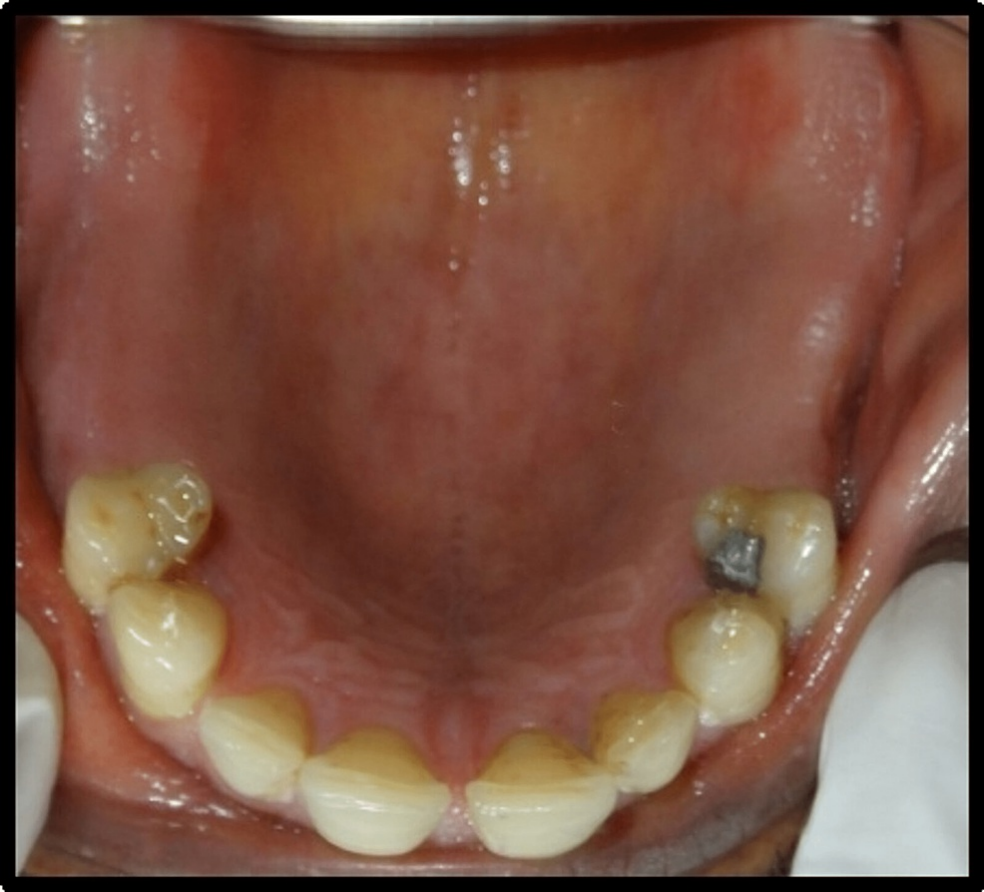
Preoperative view of intraoral maxillary arch

**Figure 2:**
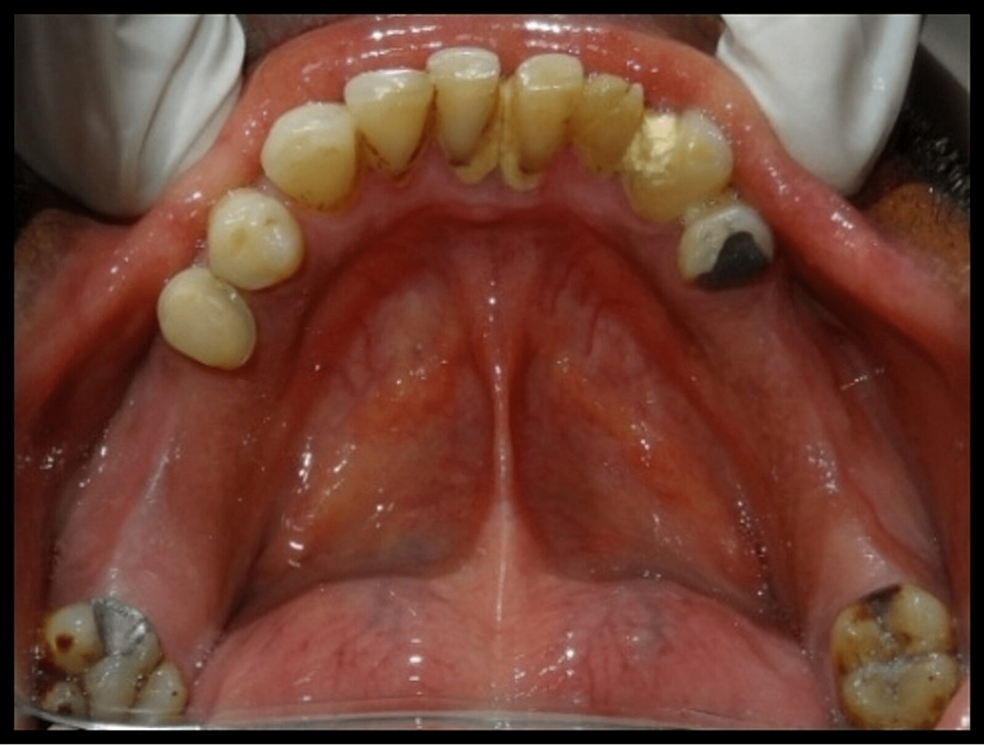
Preoperative view of intraoral mandibular arch

**Figure 3:**
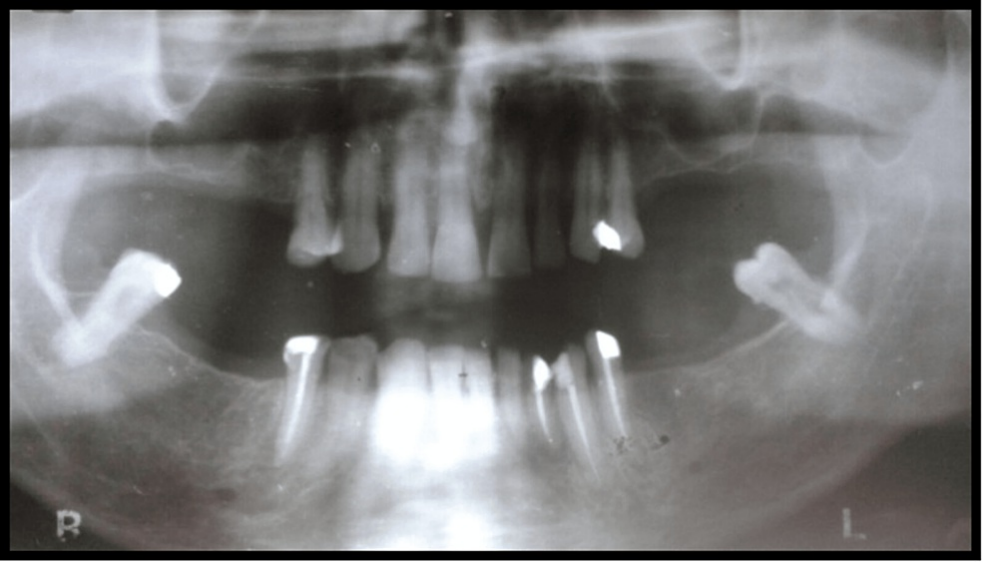
Orthopantomogram

A 45-year-old male patient reported missing maxillary and mandibular posterior teeth. The patient was partially edentulous for the past six months. On intraoral examination, the patient had bilateral missing maxillary second premolar, first molar, and second molar (Figure 1). The patient also had missing teeth in the mandibular arch in relation to the right mandibular second premolar, first molar, and second molar, and left mandibular first and second molar (45,46,47,36,37) (Figure 2). Preoperative orthopantomogram was made to evaluate the condition of the remaining teeth (Figure 3). Radiographic examination revealed root canal-treated teeth in relation to right mandibular lateral incisors, canine, and first premolar, and also root canal-treated left mandibular second premolar. The patient also has restored teeth in relation to 13 and 14.

**Figure 4:**
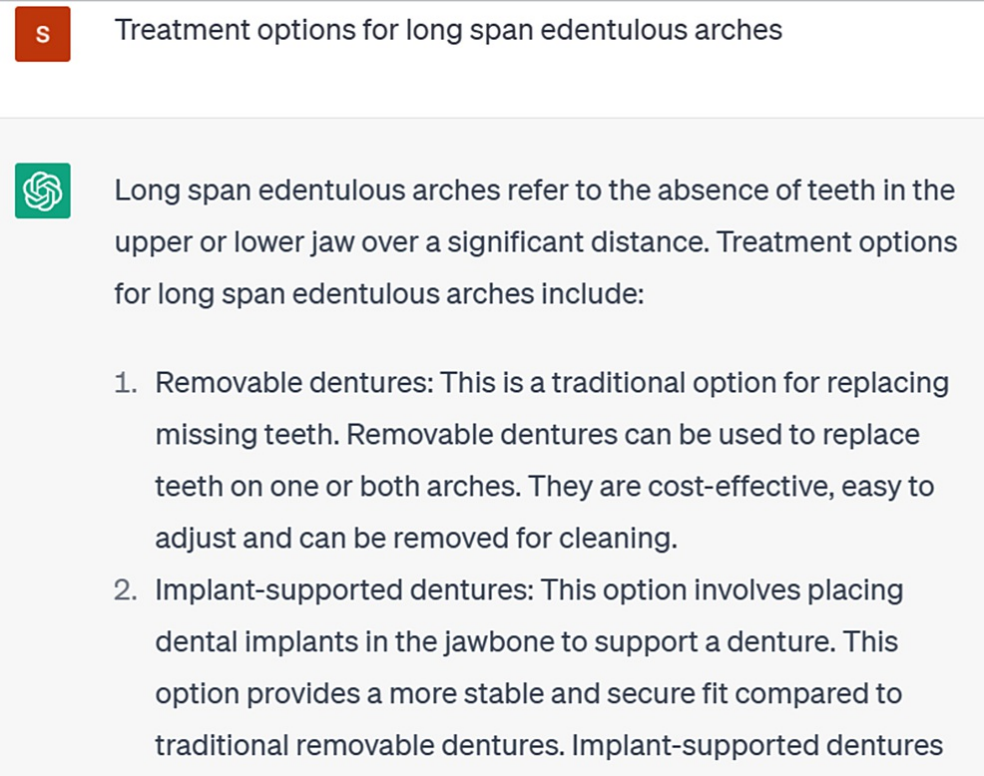
Prompts of ChatGPT for the case GPT: Generative Pre-trained Transformer Courtesy: ChatGPT

After complete radiographic and clinical examination, a prosthetic treatment plan was made. When this clinical situation was questioned in ChatGPT, it gave various treatment options in terms of dentures, partial dentures, over dentures, bridges, and implants-supported prosthesis. It concluded that the specific treatment plan for rehabilitating a long-span edentulous arch will depend on several factors, including the location of the missing teeth, the condition of the remaining teeth and gums, the patient's overall oral health, and the patient's personal preferences (Figure 4).

**Figure 5:**
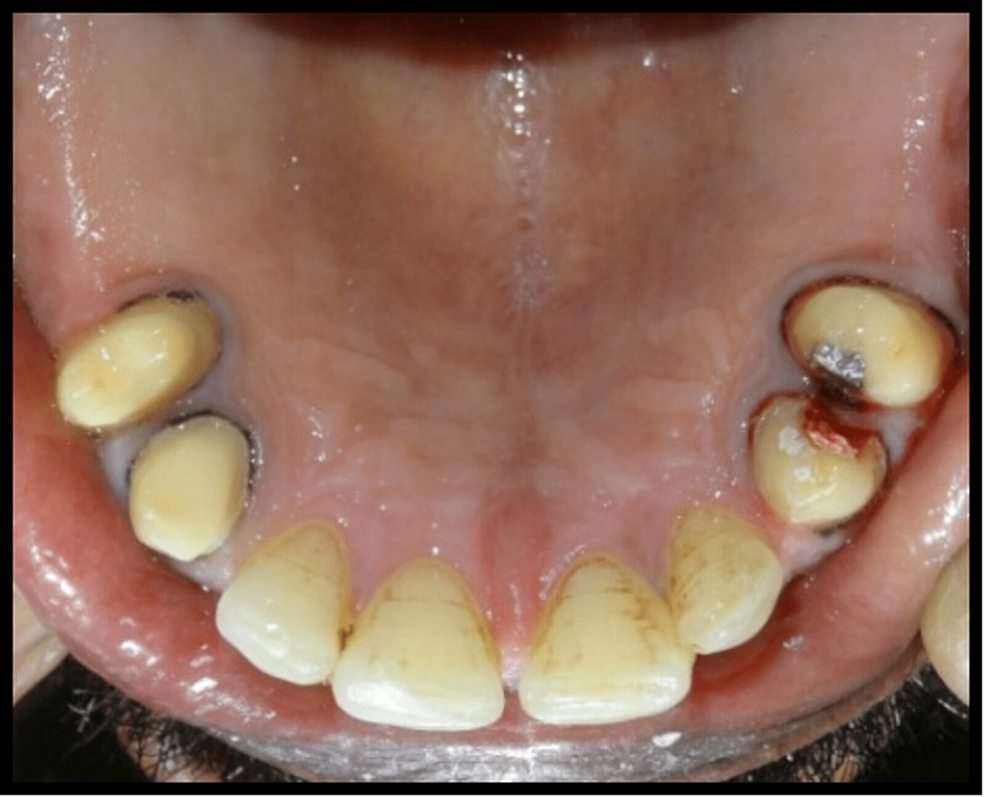
After tooth preparation (1,3,23,14,24)

**Figure 6:**
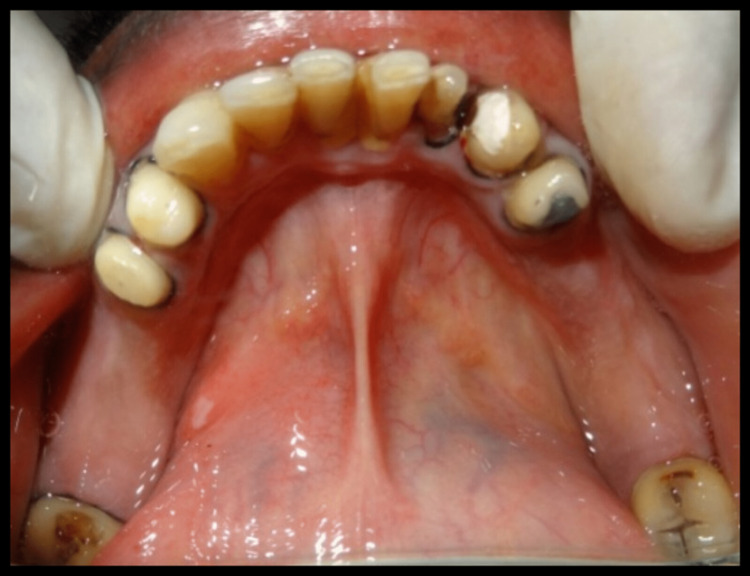
After tooth preparation (34,35,38,43,44,48)

**Figure 7:**
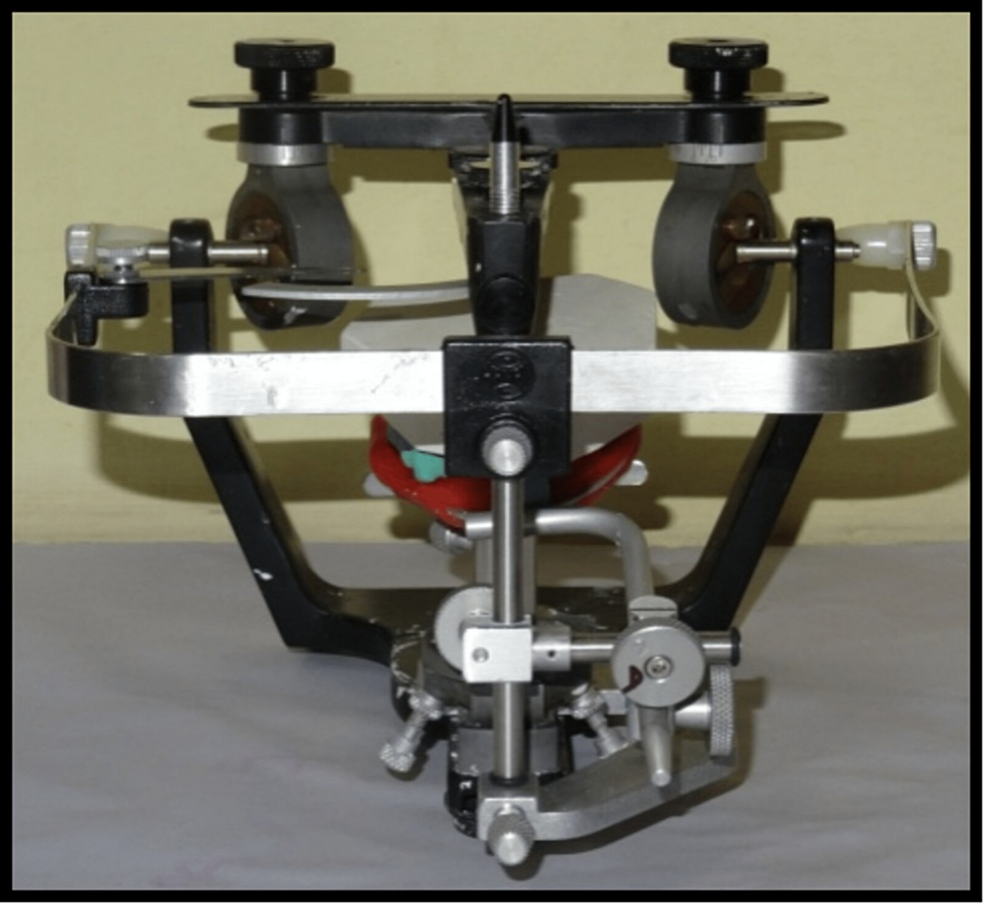
Facebow transfer

Cast partial denture with extra-coronal precision attachment was planned in the maxilla and metal ceramic fixed partial denture prosthesis in the mandibular arch. Primary alginate impression was made using Alginate (Tropicalgin, Zhermack, Italy) and a diagnostic primary cast was articulated on a semi-adjustable articulator (The Hanau Wide-Vue, Whip Mix Corporation, Louisville, USA) using centric record, and a facebow (The Hanau Spring-Bow, Whip Mix Corporation, Louisville, USA) transfers to evaluate the interarch space. The teeth considered ideal to be taken as abutments were 13,14,23,24,34,35,43,44. Tooth preparation was done in relation to 13,14,23,24,34,35,38,43,44,48 (Figures 5,6). Preparation of abutment tooth were checked for parallelism and definitive impression were made using putty and light body wash impression material (Aquasil soft putty/ regular set, Dentsply, Germany) and the second master model was made. This model was to be used for fabrication of the cast partial superstructure.The interarch space was evaluated from the primary cast which was mounted with facebow transfer (Figure 7).

**Figure 8:**
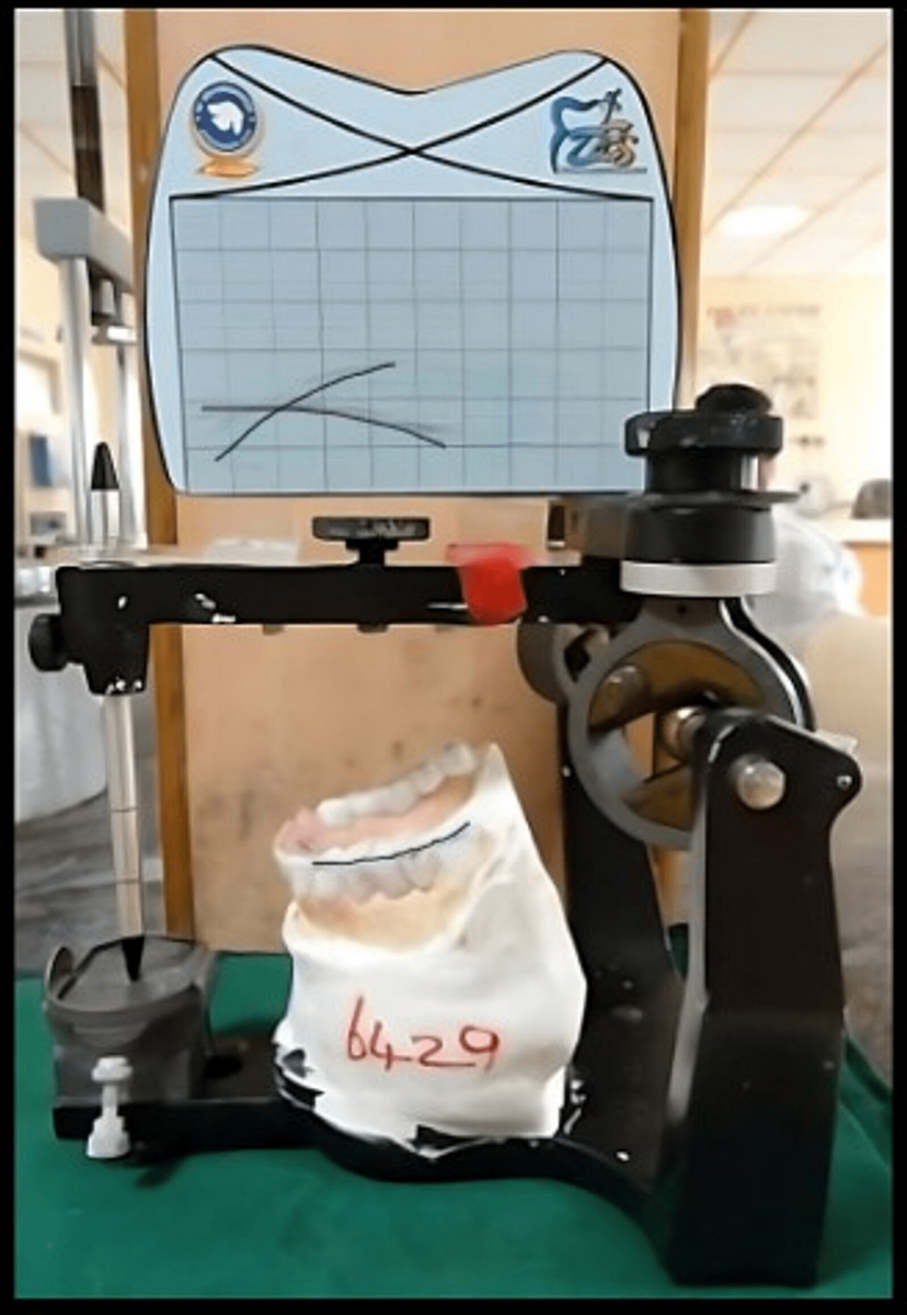
Occlusal Analysis (Broadrick Occlusal Plane Analyzer)

**Figure 9:**
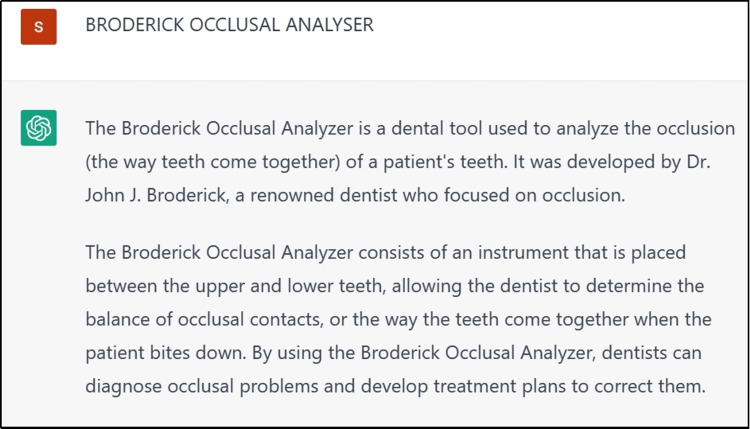
Prompts from ChatGPT GPT: Generative Pre-trained Transformer Courtesy: ChatGPT

**Figure 10:**
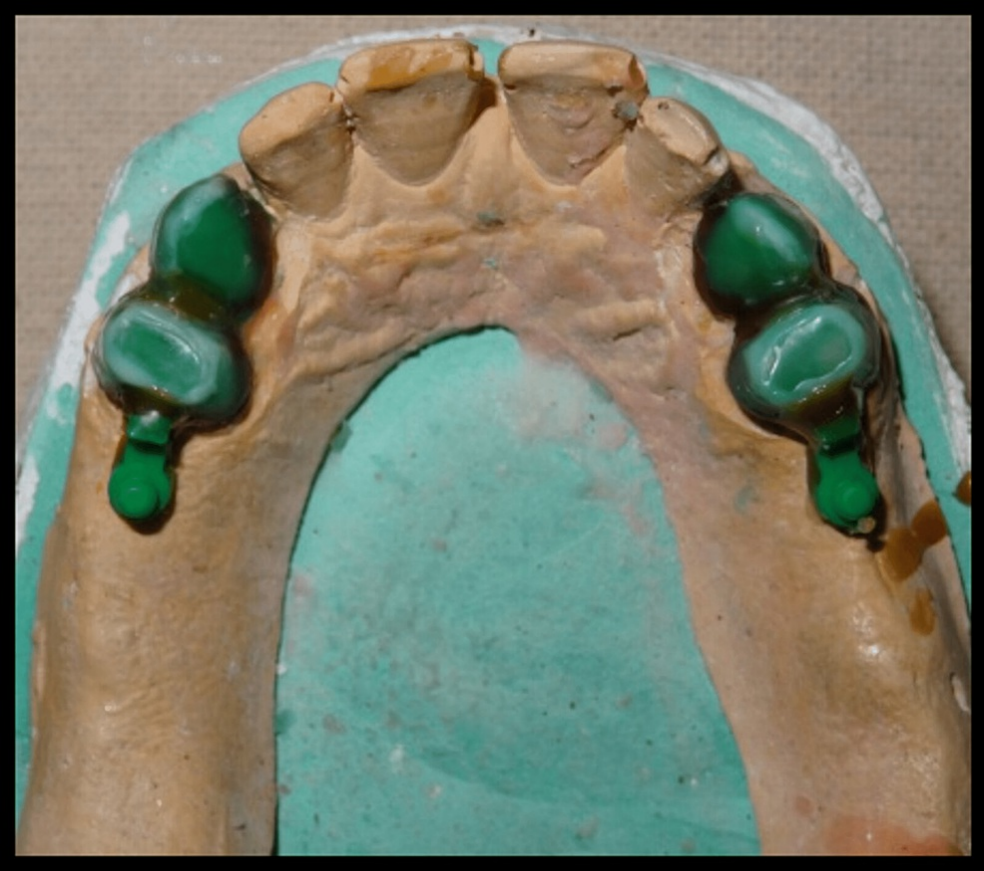
Wax pattern

**Figure 11:**
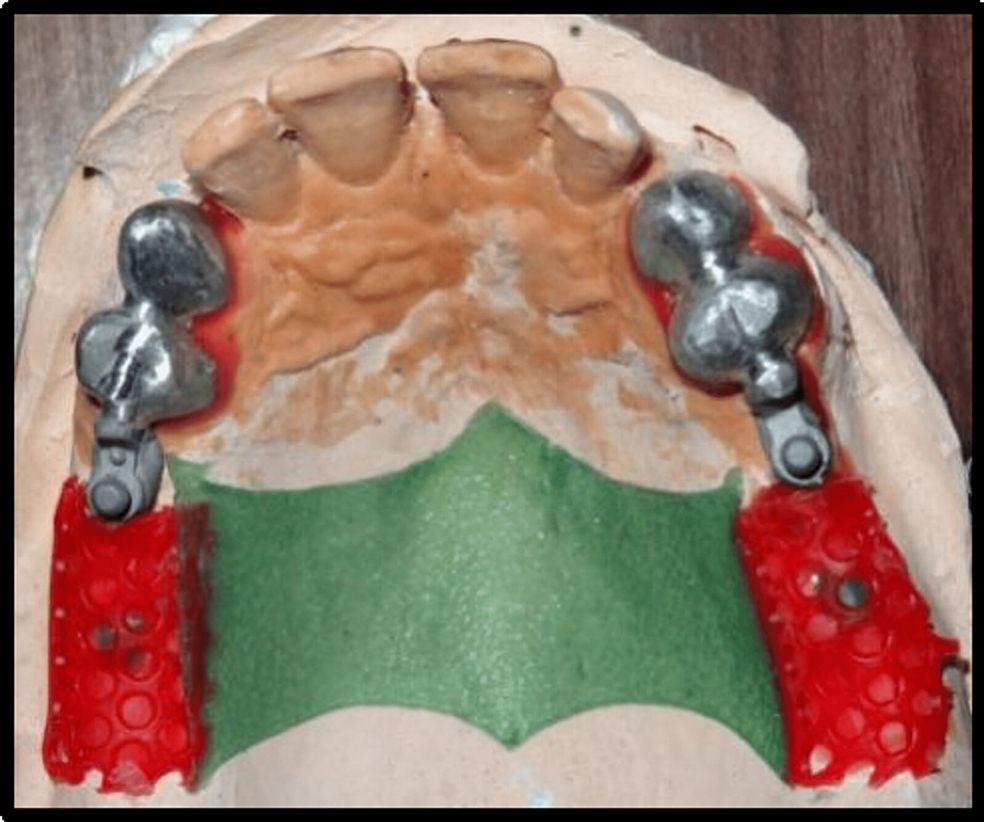
Metal copings with attachment

**Figure 12:**
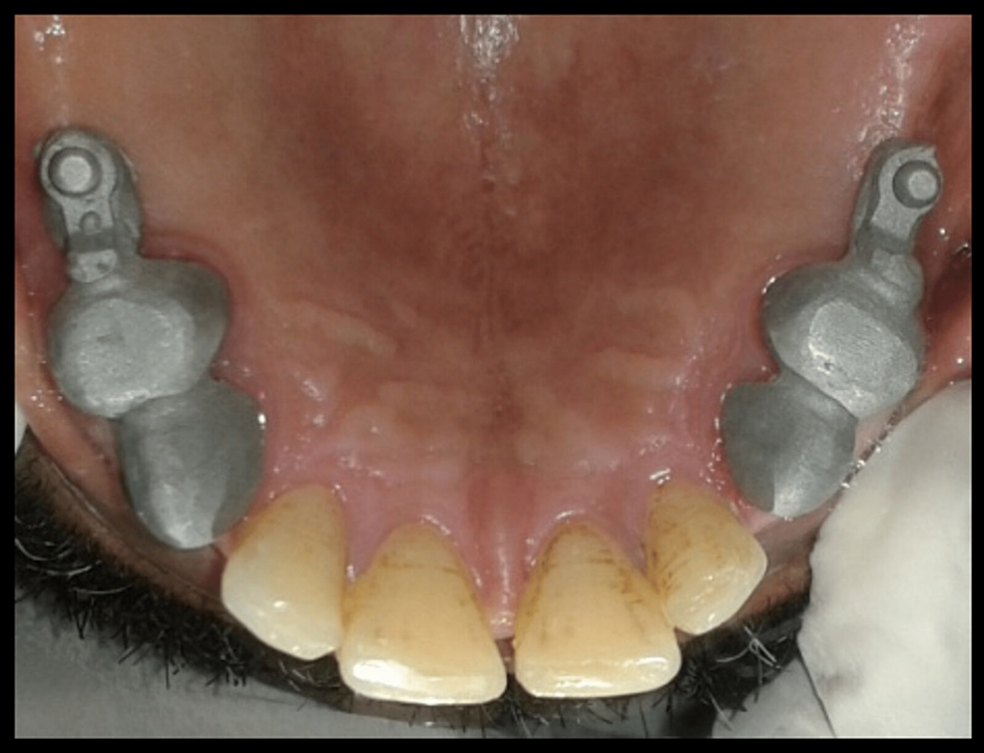
Metal try-in with attachment in the maxillary arch

**Figure 13:**
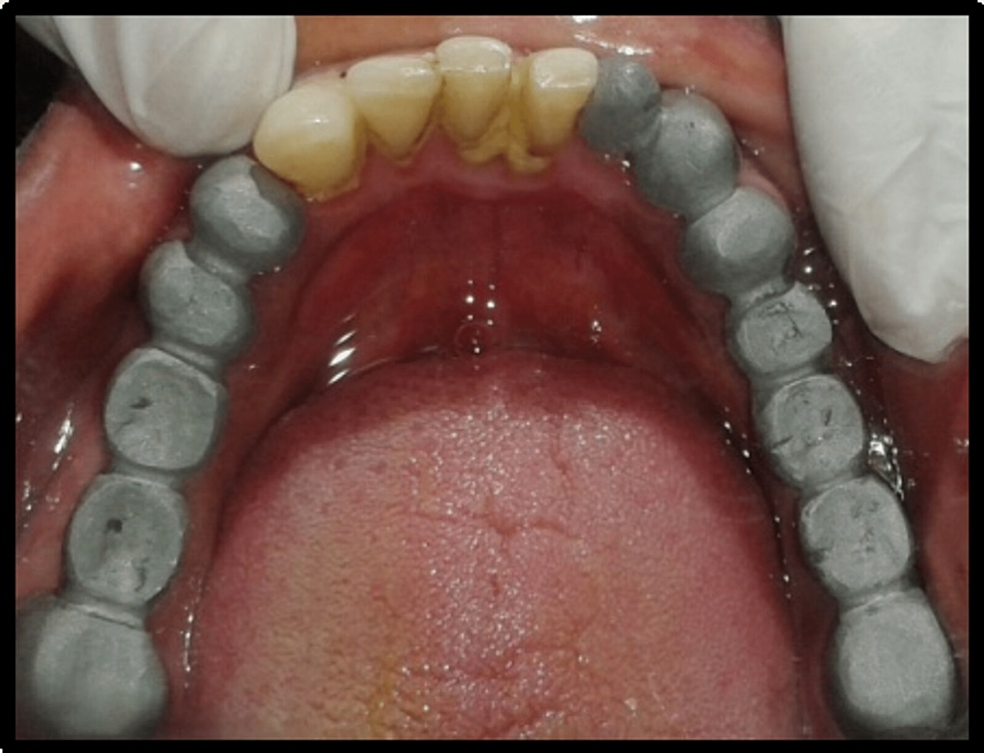
Metal try-in with attachment in the mandibular arch

The prepared tooth was temporized using temporary luting system (Protemp). When searching regarding occlusal plane analysis, information received from ChatGPT was recorded (Figures 8,9). Posterior occlusal plane was determined by Broadrick Occlusal Plane Analyzer. Posterior tooth were prepared and wax up of the prepared tooth was done in the mandibular cast. In order to achieve better occlusal clearance and temporization, a proper putty index of mock-up wax pattern was taken and occlusal plane was analyzed using Broadrick Occlusal Plane Analyzer (Figure 8). In this technique, with the help of a compass, two lines are scribed, which produces two intersecting arcs with the radius of 4 inches, where the centers of rotation is considered to be at the tip of mandibular canine and the distobuccal cusp tip of the mandibular second molar. In Broadrick Occlusal Plane Analyzer, the point where two arcs meet is considered as point of bisection, which represents the centre of rotation, and it helps in determining the mandibular occlusal plane in posterior full-mouth rehabilitation cases [3]. Maxillomandibular relation and correct vertical dimension were taken. The cast was poured and wax pattern for metal ceramic crowns with Rhein 83 attachment was fabricated. The terminal abutments selected in this case includes 13,14,23,24, to which the Rhein 83 attachments were planned to be attached to their distal ends (Figures 10,11). Articulation spaces and bulkiness were evaluated in order to proceed with optimal positioning of attachment using proper parallelometer mandrel. The Rhein 83 attachment were attached to the wax pattern of both 14 and 24 and parallelism was verified with the help of Ney Surveyor. The cast partial framework was waxed up, which was then cast using a base metal alloy (cobalt-chrome). Casting of the whole wax pattern along with the Rhein 83 attachment was carried out. Joint crowns were fabricated with the attachments in the laboratory and the trial of the same was done to check the exact fit of the crowns. Metal try in of the casted crowns with the attachments were done in the maxillary and mandibular arch analysing the fit of the metal prosthesis (Figures 12,13). After metal try in, ceramization followed by finishing and polishing of the final prosthesis was done. The final prosthesis was cemented using Type I glass ionomer luting cement (GC FUJI 1, GC Corporation, Tokyo, Japan).

**Figure 14:**
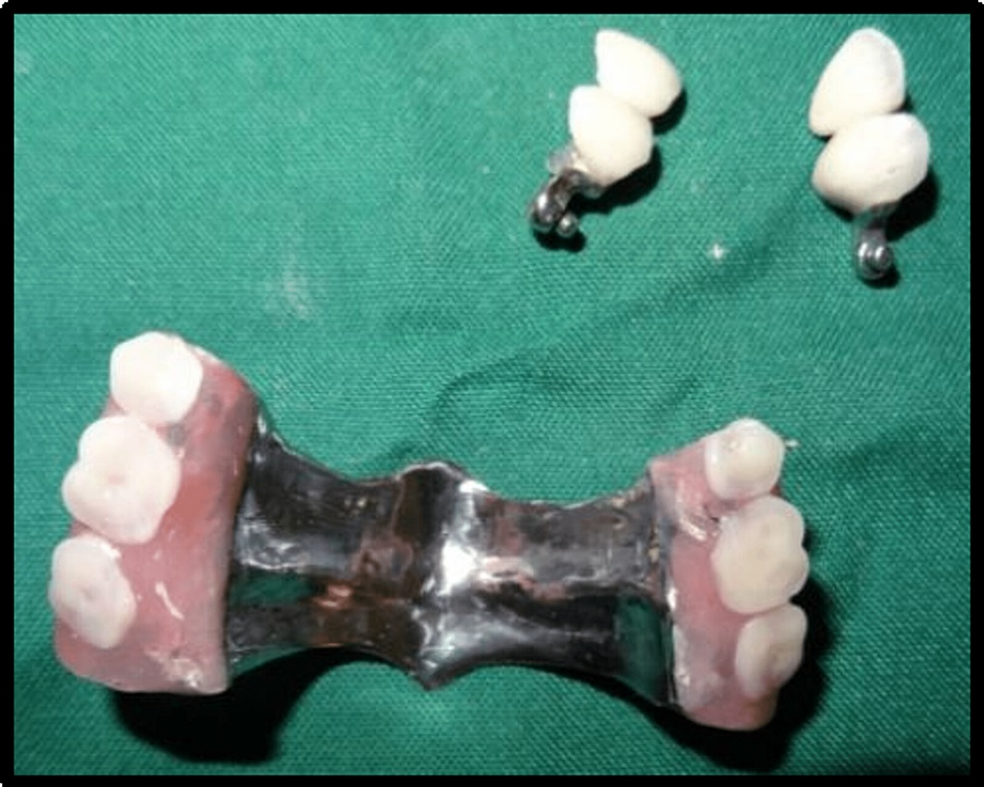
Processed cast partial denture with attachments

**Figure 15:**
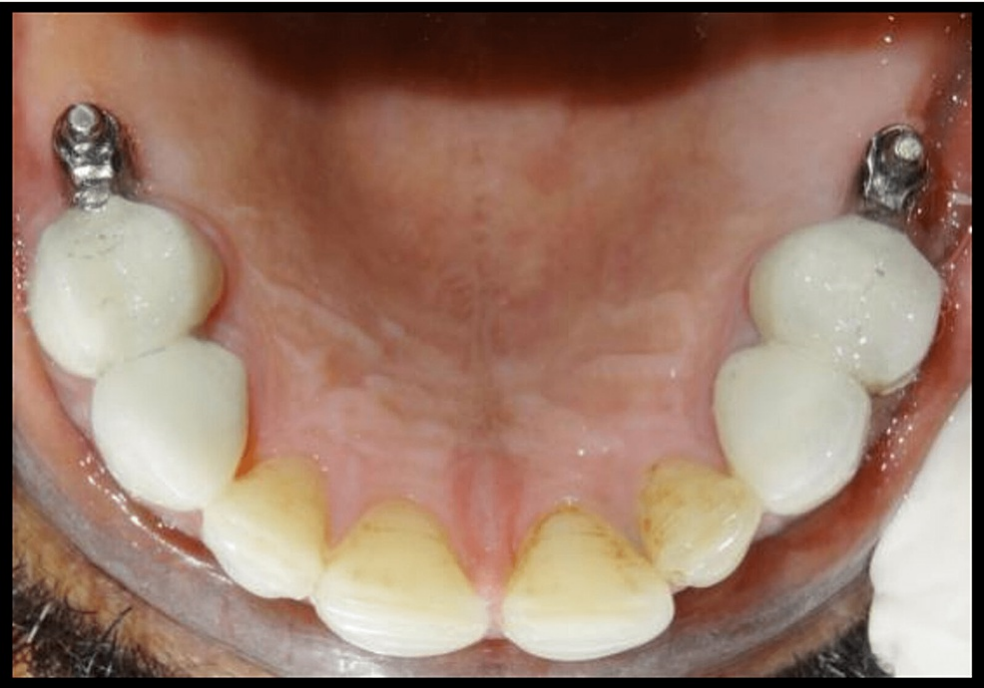
Metal ceramic crowns with attachment

**Figure 16:**
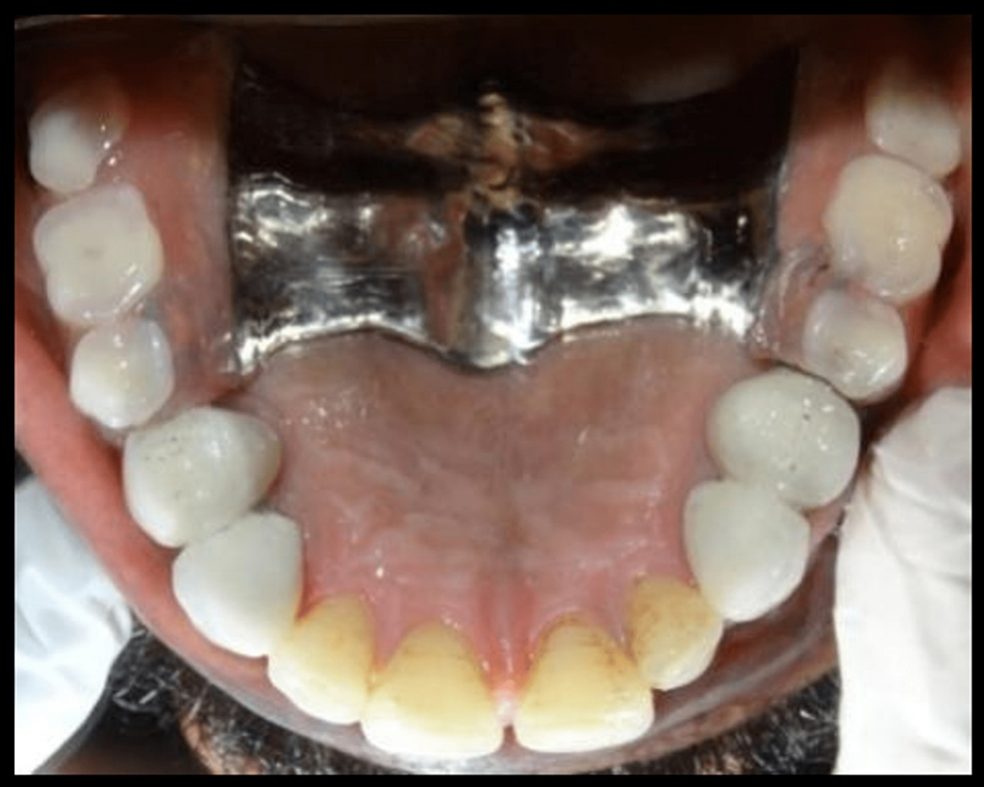
Intraoral maxillary cast partial denture

**Figure 17:**
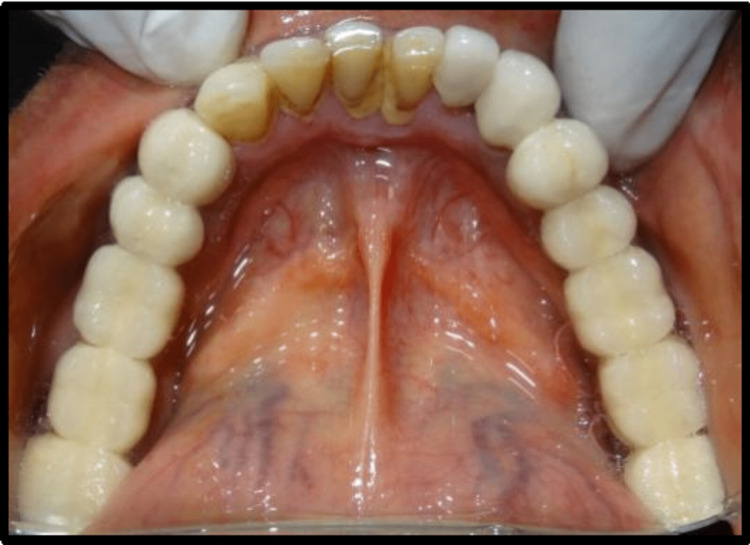
Intraoral metal ceramic crowns in mandibular arch

**Figure 18:**
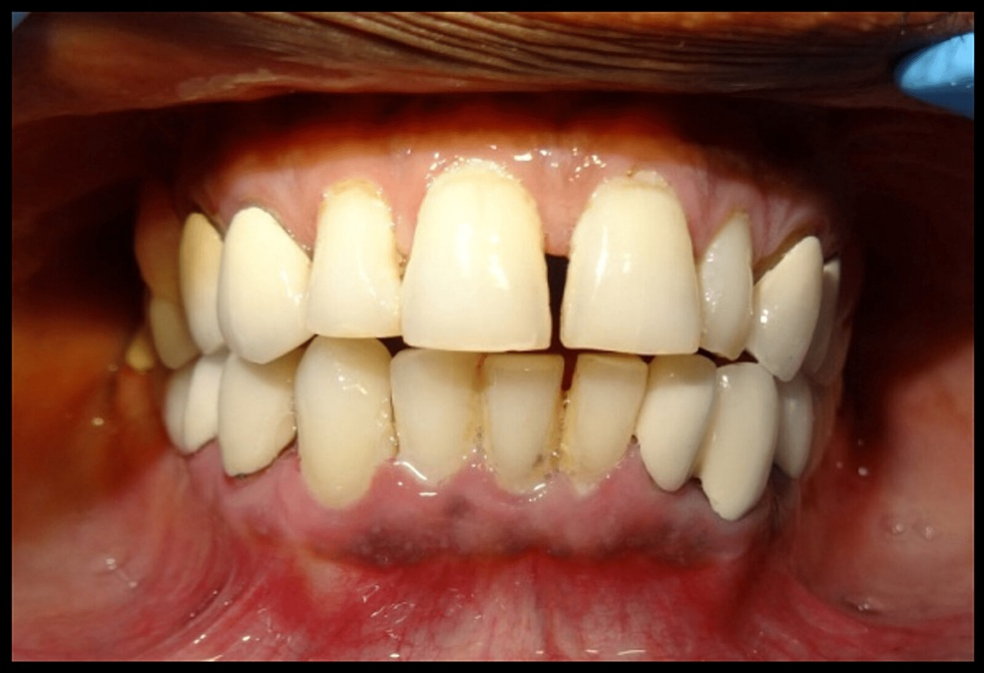
Final prosthesis

Polyvinylsiloxane impression material was used taking impression of cast partial denture framework and the cast was poured using type IV gypsum material and designing of cast partial denture framework was done. Cast partial denture with attachment was fabricated and try in of the metal framework trial was done intraorally to check the accuracy and stability of framework, following which jaw relation and teeth arrangement was carried out. The trial denture were acrylized and cast partial denture framework is finished and polished (Figure 14). Finished prosthesis was seated in patient's mouth and cementation of crowns was done using Type I Glass Ionomer cement (GC Fuji) (Figure 15). Attachments are protected with a thin layer of petroleum jelly, which acts as lubricant and helps in easy removal from cast partial denture after porcelain fused metal crowns with attachments have been seated. Complete seating of finished maxillary combined prosthesis with extracoronal castable distal extension precision attachment was evaluated clinically, and mandibular complete denture was also seated in the patient’s mouth (Figures 16,17). The patient was recalled after 24 hours for post insertion review (Figure 18).

 

## Discussion

In rehabilitation of partially edentulous cases, it is a common thought that in cases with poor support for fixed restorations, it is advisable to avoid extraction if they can support a denture. Tooth-supported overdentures enables better stress distribution, thereby maintaining the contour and integrity of residual ridge [4]. However, the rehabilitation of edentulous arches using cast partial dentures and precision attachments can provide patients with an effective and long-lasting solution for missing teeth. Precision attachments can be used to provide greater stability and retention than other types of connectors. In certain cases, the terminal abutments act as fulcrum while functional movements of removable partial denture as well as retentive clasps exert force on abutment teeth which may jeopardize the periodontium of these teeth [5,6]. When these uses of precision attachment were texted in ChatGPT, it answered in a broad manner and it explained precision attachments as specialized components of dental prosthetics that allow for a secure and stable connection between various dental devices, such as dentures, partial dentures, and bridges. 

The precision attachment provides stress-directing attachment design, and the forces are equally distributed between the abutment teeth and residual ridge. As commented by ChatGPT, it provides esthetic and functional solution, allowing for a seamless integration of the prosthetic devices with the surrounding natural teeth. This adds advantages like comfort, satisfaction, and chewing ability. The advantage of precision attachment includes balance between function as well as esthetic appearance of partial dentures in terms of stability and cosmetic appearance of the prosthesis. Precision attachments are considered as connecting components between fixed and removable prosthesis. These attachments works on the basis of stress breaking philosophy, which facilitates some amount of vertical movement which reduces the stress on abutment. Feinberg concluded from his studies that precision attachments have the characteristic of dissipating the destructive lateral forces as they are free moving and passive [7,8]. Thomas Forde, based on the principles and practice of oral dynamics, stated that forces that are concentrated vertically direct the dentinal supply to the periodontal structures, whereas rocking forces produces degeneration of underlying tooth structures [7]. Thus, the tissue that is present under a passive attachment is pink and in healthy condition due to vertically directed physiologic stimulation during function.

An attachment is a mechanical device which acts as a retainer consisting of metal receptacle and closely fitting part. It contains two components, namely, male component and female component. The female component, also called as matrix, is present in the crown structure of abutment teeth, and the male component, also called as patrix, is present attached to the pontic or denture framework. Most of extracoronal attachments produce omni polar motion, which shows high resiliency while in function. Hence, it requires multiple paths of placement for the prosthesis. The major highlight of using the attachments is that the point of application of force to the tooth is more apical when compared to incisal or occlusal rest, thereby shortening the lever arm as well as the torquing forces [9]. They also provide better cross-arch stabilization and transmission of forces when compared to components like clasps, but again, it depends on the amount of guiding surfaces, design and adaptation of the framework along with type of attachment. El Charkawi and El Wakad concluded from their study that precision attachments (metal-alloy and plastic inserts) helps in preserving the supporting teeth and alveolar bone ridges when at least two abutments are splinted [10]. Holst et al. cited that prolonged ridge resorption, variation in salivary flow and composition, and occlusal discrepancy may affect the long-term success of precision attachment, which makes it difficult to conclude its longevity through in vitro results [11,12].

## Conclusions

It is necessary to preserve the ailing teeth through attachment-retained prosthesis, which can be either fixed or removable. It is also considered as alternate mode of treatment like extraction and complete denture. Combinations of fixed and removable partial dentures with evolving new types of precision/semi-precision attachments is considered to be one of the preferred treatment options among non-affordable and contraindicated implant patients. Through this attempt of using ChatGPT for writing this case report, we appreciate the effectiveness and efficiency of ChatGPT in providing high-quality information. At the same time, it provides more general information, which is considered to be one of the disadvantages. However, long term use of ChatGPT in medical and dental fields are yet to be proved.

## References

[1] Burns DR, Ward JE (19901). A review of attachments for removable partial denture design: part 1. Classification and selection. Int J Prosthodont.

[2] Burns DR, Ward JE (1990). A review of attachments for removable partial denture design: Part 2. Treatment planning and attachment selection. Int J Prosthodont.

[3] Bedia SV, Dange SP, Khalikar AN (2007). Determination of the occlusal plane using a custom-made occlusal plane analyzer: a clinical report. J Prosthet Dent.

[4] Tallgren A (2003). The continuing reduction of the residual alveolar ridges in complete denture wearers: a mixed-longitudinal study covering 25 years. J Prosthet Dent.

[5] Ku YC, Shen YF, Chan CP (2000). Extracoronal resilient attachments in distal-extension removable partial dentures. Quintessence Int.

[6] Rathika R, Eswaran B, Ranjani T, Sameera Y (2018). An interdisciplinary approach to the management of partial edentulism using telescopic retainers. J Interdisciplinary Dent.

[7] Feinberg E (2011). Precision attachment case restoration with implant abutments: a review with case reports. J Oral Implantol.

[8] Feinberg E (1982). Diagnosing and prescribing therapeutic attachment-retained partial dentures. N Y State Dent J.

[9] Schuch C, de Moraes AP, Sarkis-Onofre R, Pereira-Cenci T, Boscato N (2013). An alternative method for the fabrication of a root-supported overdenture: a clinical report. J Prosthet Dent.

[10] El Charkawi HG, El Wakad MT (1996). Effect of splinting on load distribution of extracoronal attachment with distal extension prosthesis in vitro. J Prosthet Dent.

[11] Williams RJ, Bibb R, Eggbeer D, Collis J (2006). Use of CAD/CAM technology to fabricate a removable partial denture framework. J Prosthet Dent.

[12] Holst S, Blatz MB, Eitner S, Wichmann M (2006). In vitro wear of different material combinations of intracoronal precision attachments. Int J Prosthodont.

